# Multi‐center evaluation of the highly sensitive Abbott ARCHITECT and Alinity thyroglobulin chemiluminescent microparticle immunoassay

**DOI:** 10.1002/jcla.24595

**Published:** 2022-07-15

**Authors:** Carol Evans, Johannes Lotz, Maria Bhandari, Rowan T. Hellier, Xiao Yan Wang, Rosemarie Lott, Karl J. Lackner, Robert Müller, Vathany Kulasingam

**Affiliations:** ^1^ Department of Medical Biochemistry & Immunology University Hospital of Wales (UHW) Cardiff UK; ^2^ Institute of Clinical Chemistry and Laboratory Medicine University Medical Center (UMC), Johannes Gutenberg University Mainz Mainz Germany; ^3^ Abbott Diagnostics Abbott Park Illinois USA; ^4^ Department of Clinical Biochemistry, Laboratory Medicine Program University Health Network (UHN) Toronto Ontario Canada; ^5^ Abbott GmbH Wiesbaden‐Delkenheim Germany; ^6^ Department of Laboratory Medicine and Pathobiology University of Toronto Toronto Ontario Canada

**Keywords:** anti‐Tg, method comparision, Thyroglobulin

## Abstract

**Background:**

Thyroglobulin (Tg) is an essential part for the management of patients with differentiated thyroid carcinoma (DTC) after thyroidectomy. Highly sensitive Tg assays are now established in clinical practice as they facilitate follow‐up of DTC patients. In this study, we evaluated the recently launched highly sensitive Abbott Tg assay for Alinity and ARCHITECT.

**Methods:**

In this three‐center study, Tg values of 447 routine patient samples, characterized for the presence of anti‐Tg, were measured with the ARCHITECT Tg assay and compared with the Roche Elecsys TgII assay. In addition, a subset of 154 samples was compared also with the Beckman Tg Access assay and another subset (*n* = 122) with Abbott Tg on the Alinity i analyzer.

**Results:**

LoQ was verified to be less than or equal to 0.1 ng/ml confirming that the Tg assay on ARCHITECT and Alinity is highly sensitive. Correlation of ARCHITECT, Alinity, and Roche was excellent with a slope between 0.9 and 1.1 and a correlation coefficient >0.98. Correlation to Beckmann Tg was also very good, but the differences in absolute values were significant (slope: 1.477).

**Conclusions:**

The Abbott Thyroglobulin assay, which is standardized to CRM‐457, demonstrated a high correlation to the Roche and Beckman Tg assays, though good agreement of absolute values was only observed between Abbott and Roche. Strength of correlation and slope were not affected by the presence of anti‐Tg indicating that all assays included in the study have a similar susceptibility to anti‐Tg.

## Graphical abstract

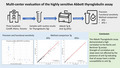

## 1 BACKGROUND

Differentiated thyroid cancer (DTC) is the most frequent endocrine malignancy. Standard treatment of DTC is thyroidectomy plus, for high risk cases, radioactive iodine ablation therapy, and pharmacological suppression of TSH. The prognosis of DTC is very good for patients without risk factors at diagnosis, like metastasis, with 5‐year survival rates >95%.^1^

Thyroglobulin (Tg) measurement is an essential diagnostic element of the follow‐up and management of patients with differentiated thyroid cancer (DTC), as the 660 kDa glycoprotein is exclusively produced by benign or well‐differentiated malignant thyroid cells. Tg is the matrix for synthesis and storage form of thyroxine (T4) and triiodothyronine (T3) within the thyroid follicles. Small amounts of Tg (approximately up to 75 ng/ml) are detected in the serum in healthy individuals and the concentration of Tg in the blood reflects thyroid mass, thyroid injury, and TSH receptor stimulation.^2^ Due to the overlap of elevated serum Tg from benign conditions such as disordered thyroid growth (goiter), increased thyroid activity (Graves' disease), or glandular destruction (thyroiditis) and DTC,^3^, ^4^, ^5^ Tg is used almost exclusively for follow‐up of DTC patients after treatment. In these patients, even very low amounts of Tg indicate persistence or recurrence of the disease.^1^

To achieve a good clinical sensitivity for detection of recurrence of DTC, Tg assays must have the capability to detect very low Tg concentrations. This is achieved through improved assay design by highly sensitive, second‐generation, immunometric Tg assays with a functional sensitivity (FS) of ~0.1 ng/ml. The significantly lower limit of quantitation compared to liquid chromatography–tandem mass spectrometry or radioimmunoassays allows detection of very low Tg concentrations and obviates the need for Tg stimulation by recombinant TSH injections to detect minimal amounts of thyroid tissues in most patients, which had to be performed for less sensitive first‐generation assays.^6^ This also results in a simplified and more cost‐efficient patient care due to direct access to patient samples.

One challenge in clinical practice, in addition to the required FS, is the presence of anti‐Tg autoantibodies as immunometric assays have been described to be susceptible to anti‐Tg autoantibodies interference causing an underestimation of the thyroglobulin concentration.^7^ It is therefore recommended to determine quantitative anti‐Tg with every measurement of Tg, especially as DTC patients have a higher anti‐Tg seroprevalence of 20%–30% compared to about 10% of the general population; though the effect of interference might be different between assays and manageable in clinical practice for most patients.^8^, ^9^, ^10^

In addition to Tg antibody interference, lack of comparability between different immunometric assays is another concern. Even though traceability of modern high sensitivity Tg assays to BCR® 457 Certified Reference Material has significantly reduced inter‐method variability to about 30%,^6^ assay results should not be used interchangeably, and according to most guidelines it is recommended not to switch the assay for monitoring DTC patients, at least not without re‐baselining, that is, dual measurement and reporting for a certain time span.

In this multi‐center study, we evaluated the analytical performance of the recently launched Abbott second‐generation Tg assay which is available for Alinity i and ARCHITECT i systems for assay performance like precision and limit of quantitation and comparability of results to Roche and Beckman Tg assays for samples negative and positive for anti‐Tg.

## 2 METHODS

### 2.1 Samples and reagents

Three different centers participated in this study. For all 3 laboratories, unidentified patient specimen leftover material was used and studies were conducted according to institutions ethical guidelines. Mostly fresh samples from routine Tg requests were used. In addition, frozen samples (Alinity i) and precision and limit of quantitation patient pools and controls were used.

Samples were measured on Abbott ARCHITECT i2000SR at three sites and Alinity i analyzer at one site using respective Tg and anti‐Tg reagents, calibrators, and controls according to manufacturer's instructions. For method comparison, samples were measured on a Roche cobas 411 at all three sites and on Beckman UniCel DxI 800 at one site using respective reagents, calibrators, and controls. Anti‐Tg status was determined using Alinity i anti‐Tg assay (UMC) or ARCHITECT anti‐Tg (UHN, UHW).

### 2.2 Precision

Precision was verified at three laboratories for ARCHITECT and one site for Alinity using a five‐day precision protocol per CLSI EP5A3 guideline. In total, 20 different samples, 17 sample pools, and 3 quality controls covering the whole measurement range were assayed.

### 2.3 Limit of quantitation (LoQ)

The manufacturer's lower LoQ defined as functional sensitivity using 20% CV precision was verified at all three laboratories for Architect and at one site for Alinity with at least two different plasma pools with 5 replicates each day for 3 days using one reagent lot on one instrument. LoQ was regarded as acceptable if concentrations were at or below 0.1 ng/ml for ARCHITECT or 0.09 ng/ml for Alinity at a CV of less than 20%.

### 2.4 Method comparison

For method comparison, samples were selected to represent the ARCHITECT and Alinity Tg analytical measurement range and results for ARCHITECT, Alinity, Beckman UniCel DxI 800, and Roche Elecsys Tg assay were obtained. Samples were also classified for the presence of anti‐Thyroglobulin antibodies using Abbott anti‐Tg assay. Samples negative and positive for anti‐Tg were analyzed separately to assess for different effects of anti‐Tg presence on Tg results.

### 2.5 Statistical analysis

All statistical analyses were carried out using Analyse‐it version 5.80.02 (Analse‐it Software, Ltd).

## 3 RESULTS

### 3.1 Precision and limit of quantitation

The mean, standard deviation, and CV of each analyte and its respective levels is shown in Table 1. The total CV was within 10% for ARCHITECT for concentrations >0.19 ng/ml, meeting the manufacturers' claimed performance.

**TABLE 1:** Precision data

Site	Analyzer	Material	Mean (ng/ml)	SD (ng/ml)	%CV
UHN	ARCH‐1	PP	0.09	0.01	16.58
UHN	ARCH‐1	PP	0.11	0.01	13.09
UHN	ARCH‐1	PP	0.14	0.02	15.47
UHN	ARCH‐1	PP	0.19	0.01	7.78
UHN	ARCH‐1	QC low	1.03	0.03	3.28
UHN	ARCH‐1	QC med	7.63	0.13	1.68
UHN	ARCH‐1	PP	62.96	1.14	1.80
UHN	ARCH‐1	QC high	337.82	6.72	1.99
UHW	ARCH‐2	PP	0.08	0.01	15.02
UHW	ARCH‐2	PP	0.74	0.03	4.24
UHW	ARCH‐2	QC low	0.99	0.03	2.85
UHW	ARCH‐2	QC med	7.46	0.13	1.75
UHW	ARCH‐2	QC‐1	11.47	0.30	2.59
UHW	ARCH‐2	PP	47.41	1.31	2.77
UHW	ARCH‐2	QC‐3	153.57	3.27	2.13
UHW	ARCH‐2	QC high	342.06	5.90	1.72
UMC	ARCH‐3	PP	0.10	0.02	16.14
UMC	ARCH‐3	PP	0.10	0.01	14.00
UMC	ARCH‐3	QC low	1.01	0.03	3.31
UMC	ARCH‐3	PP	3.92	0.09	2.24
UMC	ARCH‐3	QC med	7.60	0.19	2.54
UMC	ARCH‐3	PP	20.54	0.39	1.89
UMC	ARCH‐3	QC high	338.08	7.00	2.07
UMC	Alinity	PP	0.05^a^	0.005	10.9
UMC	Alinity	PP	0.09	0.01	7.00
UMC	Alinity	PP	0.52	0.016	2.69
UMC	Alinity	QC low	0.98	0.03	3.08
UMC	Alinity	PP	3.92	0.08	1.97
UMC	Alinity	QC med	7.63	0.16	1.83
UMC	Alinity	PP	21.48	0.42	1.93
UMC	Alinity	QC high	340.82	6.93	1.84

Abbreviations: PP, patient pool; QC, quality control.

^a^ Sixteen data points only.

Using the 5‐day protocol for within‐run precision highlighted precision below 3.4% for panels ranging from 1.01 to 338.08 ng/ml. For concentrations of 0.19 and 0.74 ng/ml, maximum imprecision was 7.78% and 4.24%, respectively. Imprecision results spanning a concentration range from 0.19 to 338 ng/ml for ARCHITECT from all three sites ranged from 7.78% to 1.68%; consistent with the manufacturer claimed performance data and current state of the art performance.^11^ Also, performance data were well below the respective product requirements for the assay (SD ≤ 0.06 ng/ml and ≤20 %CV for samples equal to or greater than the LoQ to less than 0.80 ng/ml, CV ≤ 7% for samples greater than or equal to 0.80 ng/ml and less than or equal to 6.25 ng/ml, and CV ≤ 10% for samples greater than 6.25 ng/ml to less than or equal to 500.00 ng/ml).

**FIGURE 1:**
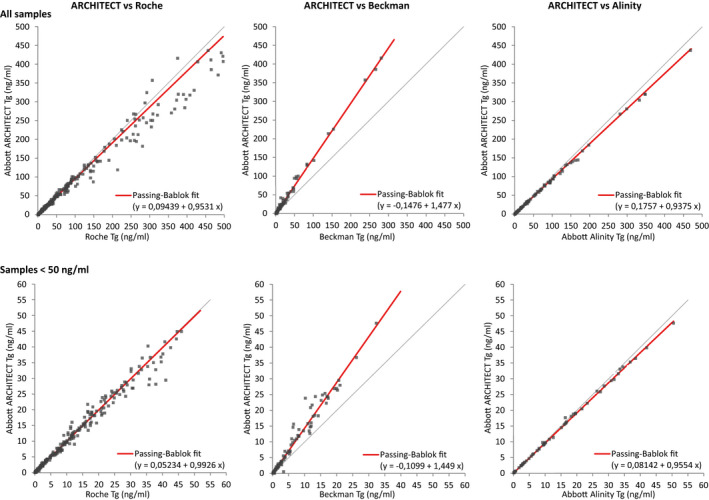
Five‐day precision profiles for Tg on ARCHITECT (three sites) and Alinity (one site). Blue lines indicate requirements for imprecision (20% CV) and concentration (0.1 ng/ml)

The LoQ defined as functional sensitivity using 20% CV criteria was at or below 0.1 ng/ml for ARCHITECT at all customer sites and <0.09 ng/ml for Alinity at one site with an observed CV of less than 20% (range: 13.09–16.58) for samples with analyte concentrations of 0.08–0.14 ng/ml for ARCHITECT and 0.09 for Alinity (CV: 7.0%; Table 1); which confirmed the IFU performance data (LoQ of 0.1 ng/ml for ARCHITECT and 0.09 ng/ml for Alinity). The precision profile of the LoQ and precision studies are shown in Figure 1.

### 3.2 Method comparison

**FIGURE 2:**
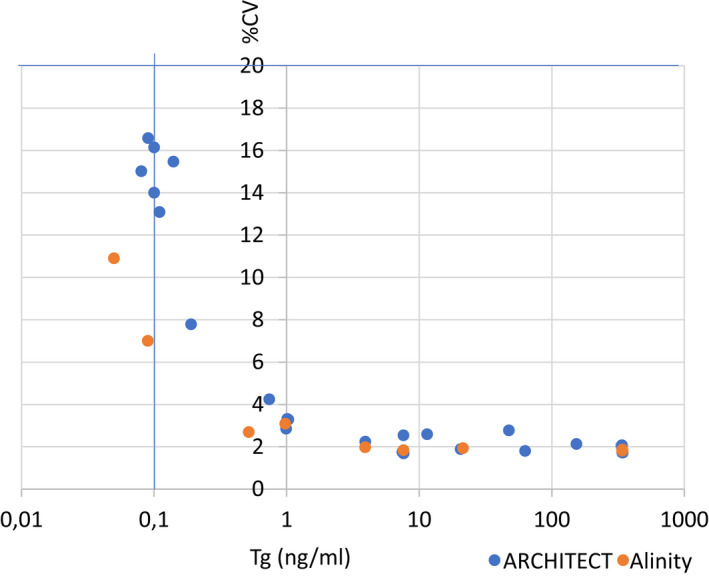
Method comparison data for Abbott ARCHITECT versus Roche, Beckman, and Alinity using Passing‐Bablok fit for anti‐Tg‐negative samples. Upper row: All samples, lower row samples up to 50 ng/ml (mean of both methods)

**FIGURE 3:**
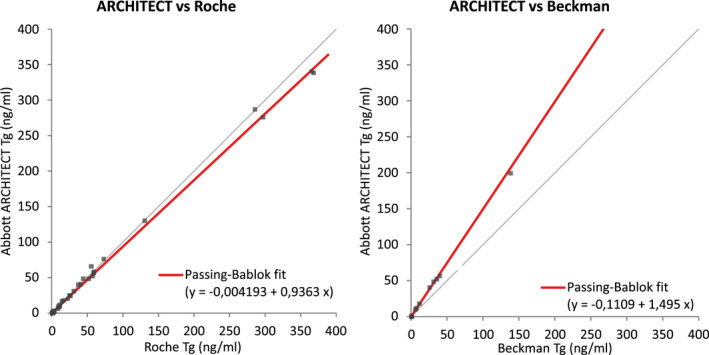
Method comparison data for Abbott ARCHITECT versus Roche and Beckman using Passing‐Bablok fit for anti‐Tg‐positive samples

Results for the method comparison are summarized in Figures 2 and 3. Only samples which were between the LoD and the upper limit of the analytical measurement range for both methods were included in the figures. Analysis including the few high concentration samples (*n* = 9, up to 25,249 ng/ml) was done but did not result in any significant differences (not shown). Samples tested negative or positive for anti‐Tg were analyzed separately (Figures 2 and 3).

Serum Tg measured using with ARCHITECT was compared with Elecsys Tg II results. In total, 447 patient samples were analyzed for ARCHITECT and Roche with 56 samples positive for anti‐Tg (cutoff 4.1 IU/ml). ARCHITECT Tg concentrations were ranging from 0.09 to 486 ng/ml for anti‐Tg‐negative and 0.09 to 341 ng/ml for anti‐Tg‐positive samples. Roche Tg values were closely correlated, with a correlation coefficient greater than 0.98 and a slope within 0.9–1.1 for anti‐Tg‐negative and anti‐Tg‐positive samples. Subrange analysis revealed that the correlation was maintained even at low concentration samples (below 50 ng/ml).

In addition, 122 samples (16 anti‐Tg positive) were analyzed on Abbott ARCHITECT versus Alinity and a close correlation of the two methods was observed (*r*: 0.999, slope: 0.94). A close correlation was also observed, when comparing ARCHITECT to Beckman (*n*: 154, 18 positive for anti‐Tg) but with a significant bias (slope: 1.477 and *r*: 0.974 for anti‐Tg‐negative samples).

Results of the ARCHITECT vs. Roche, Beckman, and Alinity method comparison are summarized in Figure 2 for anti‐Tg‐negative samples with separate plots for low concentration samples (<50 ng/ml) mean of both methods and Figure 3 for the method comparisons of ARCHITECT versus Roche and Beckman of anti‐Tg‐positive samples.

Ratio of slopes of method comparisons from anti‐Tg‐positive samples divided by the respective slopes of anti‐Tg‐negative samples were calculated in Table 2 to estimate different susceptibilities of the different assays for anti‐Tg interference (Figures und ratios for Alinity i, which uses same reagent formulation as ARCHITECT, are not shown as only data versus Roche were available).

**TABLE 2:** Comparison of method correlation between anti‐Tg‐negative and positive samples for ARCHITECT versus Roche and Beckman methods

	Slope anti‐Tg negative	Slope anti‐Tg positive	Ratio anti‐Tg+/anti‐Tg‐
ARCHITECT vs. Roche	0.953	0.936	0.982
ARCHITECT vs. Beckman	1.477	1.495	1.012

*Note:* Ratios between the methods were calculated to evaluate differences in method correlation in regard to anti‐Tg status.

## 4 DISCUSSION

In this study, the second‐generation highly sensitive new ARCHITECT and Alinity Tg assays were examined. The precision performance was in line with the manufacturer's claims for all three sites (Table 1 and Figure 1). The data for assay imprecision were well below the product requirement of 10% CV for samples ranging from 0.19 to 338 ng/ml and additionally meeting the desired specification of 7.5% analytical CV according to the EFLM biological variation database (accessed 08 Oct 2020) for a concentration range from 0.74 to 338 ng/ml and the minimum requirement of 9.6% for the 0.19 ng/ml sample for ARCHITECT. For Alinity samples from 0.52 to 341 ng/ml showed a precision of less than 3.1% meeting the optimum specification of 3.8%. Of note, the precision of 7.00% on Alinity of the sample with a concentration of 0.09 ng/ml, used for the LoQ study, was still meeting the desired analytical CV of 7.5% showing an excellent precision profile of the Tg assay on the Alinity i instrument.

The claimed limit of quantitation of 0.1 ng/ml for ARCHITECT was confirmed at all three laboratories as was the LoQ of 0.09 ng/ml for the Alinity Tg assay at one site. Both assays thus can be classified as highly sensitive with a functional sensitivity <0.1 ng/ml.^6^

The performance for precision and FS is in line with other second‐generation assays^11^, ^12^ and allows correct classification of patients for risk assessment according to the ESMO and ATA guidelines where a concentration of <0.2 ng/ml is used as threshold for excellent response to treatment.^1^, ^13^

The evaluation of Abbott ARCHITECT and Alinity Tg assay was continued with a comparison of patient samples, both anti‐Tg negative and anti‐Tg positive, with the established highly sensitive Roche Elecsys TG II and Beckman assays. For anti‐Tg‐negative samples we found a very close correlation (*R* ≥ 0.99) for ARCHITECT and Alinity as would be expected as both assays use the same formulation but also a close agreement of absolute values demonstrated by a slope 0.953 for ARCHITECT and 1.01 for Alinity (data not shown) if comparing to Roche using Passing‐Bablok regression.

Similar results were also obtained for anti‐Tg‐positive samples with *R* ≥ 0.99 and a slope of 0.936 for ARCHITECT and 1.01 for Alinity. Taken together, the results from the method comparison studies indicate that the standardization to the BCR® 457 Certified Reference Material did result in an acceptable commutability of the Abbott and Roche methods which should facilitate the adoption of the Abbott ARCHITECT and Alinity methods in this case, as only limited parallel measurements are required.

In contrast, values obtained with ARCHITECT Tg were significantly higher than the Beckman assay values with a slope of 1.477 for anti‐Tg‐negative samples and 1.495 for anti‐Tg‐positive samples, which is in line with other observations, were in contrast to the observed commutability between Roche and Abbott a lack of commutability was demonstrated for several methods standardized to BCR® 457 Certified Reference Material,^6^ which is not uncommon for immunoassays detecting complex molecules. As overall correlation was good between ARCHITECT and Beckman Tg with an *R* value of 0.97, it might still be possible to convert values using a factor, though that would require more studies.

To check for differences of possible anti‐Tg interference, we compared the slopes of anti‐Tg‐negative and positive samples for ARCHITECT versus Roche and Beckman. As shown in Table 2, the slopes were virtually identical between the two sample subsets indicating that all three assays have a similar robustness regarding potential anti‐Tg interference. We could not, however, demonstrate a higher susceptibility of the Beckman assay as described by (Rotteveel‐de^11^), which might be due to methodological limitation of our study, though it should be noted that^14^ found a slightly lesser susceptibility of the Beckman versus the Roche assay regarding anti‐Tg interference. The similar susceptibility regarding anti‐Tg, especially between Abbott and Roche, is further corroborated by the additional data of the method comparison data, though we did not assess anti‐Tg interference directly and acknowledge the poor agreement of anti‐Tg assays.^15^

The lower Tg recovery of immunometric assays in presence of anti‐Tg antibodies has been a concern of the clinical utility of these assays in anti‐Tg‐positive patients due to falsely low results.^16^ Recent work by Giovanella et al.,^12^ however, determined optimal cutoffs the Roche, Beckman, and BRAHMS for patients negative and positive for anti‐Tg. They could demonstrate, that the cutoff for anti‐Tg‐positive patients was lower than for anti‐Tg‐negative patients but well above the LoQ of 0.1 ng/ml for Roche and Beckman (0.125 ng/ml for Elecsys and 0.2 ng/ml for UniCel DxI 800). These cutoffs resulted in minimal differences of diagnostic accuracy for anti‐Tg‐positive and negative patients thus indicating that immunometric Tg assays could be used in clinical practice also for anti‐Tg‐positive patient without compromising diagnostic accuracy. It should be noted, that these results should be confirmed in larger studies before changing clinical practice, but it might be speculated that if including the Abbott Tg assay, similar cutoff values for anti‐Tg‐positive and negative patients compared with the Roche assay would likely be observed due to the close agreement in both anti‐Tg sample subsets.

Overall, this study shows that the new Abbott Tg assay is comparable to other current second‐generation assays and though that the study had the limitation that no clinical diagnosis was obtained for the patient samples, the very close agreement to the Roche method is good indication that a similar clinical performance might be expected for the Abbott Tg assay.

## AUTHOR CONTRIBUTIONS

CE, MB, KJL, JL, RM, and VK designed the study. RL, JL, XYW, RTH, MB, and RMS were responsible for data collection and management. RM and MB were responsible for biostatistics analyses. CE, MB, JL, KJL, RM, and VK were responsible for interpretation of data. RM and MB prepared the tables and Figures. RM did drafting of article. All authors contributed to revision of the article and approved it for submission.

## CONFLICT OF INTEREST

RM and MB are employees of Abbott. The other authors have declared no conflicts of interest.

## Data Availability

The data that support the findings of this study are available from the corresponding author upon reasonable request.
